# Wnt-Signaling Regulated by Glucocorticoid-Induced miRNAs

**DOI:** 10.3390/ijms222111778

**Published:** 2021-10-29

**Authors:** Henriett Butz, Katalin Mészáros, István Likó, Attila Patocs

**Affiliations:** 1 Hereditary Tumours Research Group, Hungarian Academy of Sciences and Semmelweis University, H-1089 Budapest, Hungary; butz.henriett@med.semmelweis-univ.hu (H.B.); kati.balla@gmail.com (K.M.); istvanliko@gmail.com (I.L.); 2 Department of Laboratory Medicine, Semmelweis University, H-1122 Budapest, Hungary; 3 Department of Molecular Genetics, National Institute of Oncology, H-1122 Budapest, Hungary

**Keywords:** miRNA, Wnt signaling, Cushing, adrenal, hypercortisolism

## Abstract

Glucocorticoids (GCs) are pleiotropic hormones which regulate innumerable physiological processes. Their comprehensive effects are due to the diversity of signaling mechanism networks. MiRNAs, small, non-coding RNAs contribute to the fine tuning of signaling pathways and reciprocal regulation between GCs and miRNAs has been suggested. Our aim was to investigate the expressional change and potential function of GC mediated miRNAs. The miRNA expression profile was measured in three models: human adrenocortical adenoma vs. normal tissue, steroid-producing H295R cells and in hormonally inactive HeLa cells before and after dexamethasone treatment. The gene expression profile in 82 control and 57 GC-affected samples was evaluated in GC producing and six different GC target tissue types. Tissue-specific target prediction (TSTP) was applied to identify the most relevant miRNA−mRNA interactions. Glucocorticoid treatment resulted in cell type-dependent miRNA expression changes. However, 19.5% of the influenced signaling pathways were common in all three experiments, of which the Wnt-signaling pathway seemed to be the most affected. Transcriptome data and TSTP showed similar results, as the Wnt pathway was significantly altered in both the GC-producing adrenal gland and all investigated GC target tissue types. In different cell types, different miRNAs led to the regulation of similar pathways. Wnt signaling may be one of the most important signaling pathways affected by hypercortisolism. It is, at least in part, regulated by miRNAs that mediate the glucocorticoid effect. Our findings on GC producing and GC target tissues suggest that the alteration of Wnt signaling (together with other pathways) may be responsible for the leading symptoms observed in Cushing’s syndrome.

## 1. Introduction

Glucocorticoids (GCs) are steroid hormones which regulate the various metabolic and homeostatic processes essential for life: inflammatory and immune reactions, metabolic control, fertility and reproduction, cognitive function and development. Their role is vital for the physiological function of many organs.

Although endogenous hypercortisolism is a relatively rare condition (the incidence of newly diagnosed cases varies between 0.7–3.0/1 million inhabitants/year [1,2]), synthetic glucocorticoids are widely used for their anti-inflammatory and immunomodulatory effects. GCs are essential in the treatment of chronic inflammatory diseases, after organ transplantations and in the field of oncohematology, but the harmful effects accompanying hypercortisolic periods throughout prolonged GC administration represent a serious risk for many pathological conditions.

GC signaling is mediated by the glucocorticoid receptor whose activation ultimately leads to the stimulation of the target gene’s expression [3,4]. Diverse signaling mechanisms admit glucocorticoids to control the physiological processes at the level of different organs and tissues, but it is difficult to define the “main” signaling pathways which are responsible for these processes. The most studied pathways related to GC signaling are: the GH/IGF-1 axis, TGFβ-SMAD signaling, PI3/Akt signaling, MAPK signaling, Wnt-signaling, BMP signaling and NF-κB signaling [5,6,7,8,9,10,11].

Wnt ligands are small glycoproteins which bind to the Frizzled (Fz) receptor. In the absence of a Wnt ligand, a destruction complex composed of Axin and adenomatous polyposis coli is responsible for the constant phosphorylation of β-catenin by glycogen synthase kinase 3β (GSK-3β) and casein kinase type 1. The binding of Wnt to the Fz receptor requires the presence of low-density lipoprotein receptor-related-protein 5 or 6 (LRP 5/6) as coreceptor. Wnt-Fz-LRP 5/6 complex formation leads to the membrane recruitment of the Axin complex and to the consequential suspension of the degrading complex’s operation. Escaping from phosphorylation, the free β-catenin level increases and forms complexes with the DNA-bounded T cell factor/lymphoid enhancer factor (TCF/LEF) to activate Wnt target gene expression. Wnt-pathway agonists are a large family from which the role of the Dickkopf family (Dkks), sclerostin (Sost) and secreted Frizzled-related proteins (sFRPs) were identified as important inhibitors in glucocorticoid excess [12,13,14,15].

MiRNAs, evolutionary conserved non-coding RNAs regulate various biological processes through post-transcriptional modifications of mRNAs. Their role was demonstrated in numerous physiological and pathological processes in different tissues, including the development, homeostasis and aging processes [16].

The mediating role of the miRNAs in glucocorticoid action has been extensively studied. miRNAs regulate glucocorticoid production and glucocorticoid availability, the latter being accomplished through the modulation of glucocorticoid receptors’ expression levels [17]. They also have an important role in cell response to the presence of glucocorticoids [17]. MiRNAs are implicated in the regulation of immune and inflammatory processes, through mediating glucocorticoids’ tumor-suppressive effects in hematological malignancies (and also, in the regulation of resistance to GC-induced apoptotic processes) and in fine-tuning the responses of GCs during developmental processes and through activities which require continuous remodeling (brain, bones) [17].

Beyond the physiological processes, miRNAs have been identified as the intermediaries of modified functioning during GC excess.

Hypercortisolism represents a risk factor for cardiovascular events which can be explained, at least partly, by endothelial dysfunctions. In patients with Cushing’s syndrome (CS), miRNA analysis identified the dysregulation of endothelium-related miRNAs: miRNA-17-5p, miRNA-126-3p, and miRNA-126-5p were downregulated, while miRNA-150-5p and miRNA-223-3p were upregulated compared to controls [18]. Thrombospondin-1, an adhesive glycoprotein associated with platelet aggregation, angiogenesis and tumorigenesis, was downregulated in the pituitary tissue of patients with Cushing’s disease, and this correlated inversely with miR-449c expression [19].

Using miRNAs, it seems possible to distinguish Cushing’s disease from ectopic ACTH overproduction: miR-16-5p was overexpressed in CD, but downregulated in hypercortisolism from an ectopic source, compared to healthy controls [20]. Interestingly, in a recent article, miR-122-5p was identified as a possible link between nonphysiological (externally administered) GCs and the effects caused by GC administration. Additionally, the measurement of miR-122-5p expression can be a potential marker to monitor appropriate glucocorticoid activity and to avoid overtreatment [21].

The aim of our work was to study the involvement of different miRNAs in mediating the effects of hypercortisolism and to reveal the most affected, miRNA-regulated signaling pathways during this process in the GC producing and target tissue/cell types. Our experiment consisted of three models for miRNA profiling. We investigated the changes of the miRNA expression pattern in adrenal tissues obtained from patients with hormonally active Cushing’s syndrome compared with normal adrenal tissues. Then, we examined the changes of miRNA expression in steroid-producing adrenocortical H295R cells and in hormonally inactive (hormone nonproducing) HeLa cells before and after dexamethasone treatment. The HeLa cell line was included as a general model for studying glucocorticoid effects on epithelial cells. It is the most widely used cell line model for investigating human cellular and molecular biology [22,23] and it was also reported to be a relevant model for studying glucocorticoid-related effects due to its epithelioid origin and as it expresses glucocorticoid receptor [24,25,26,27,28,29]. Following miRNA-profiling in these experiments, pathway analysis and literature data mining were performed to investigate the relevant miRNA-pathway interactions related to glucocorticoid excess. Furthermore, we analyzed the transcriptome data of GC producing and target organs/cell types (bone tissue originated from Cushing patients and primary bone, synovial fibroblast, subcutaneous and omental adipose tissue, skin and brain cells) upon GC effect. We performed tissue-specific target prediction and gene set enrichment analysis in order to investigate the changes in Wnt signaling.

## 2. Results

In all three models, the expression of 265 individual miRNAs were measured. Of these 265 miRNAs, in adrenal adenoma tissues five miRNAs showed significantly altered expression compared to normal adrenal tissue, in H295R and HeLa cells expression of six and eight miRNAs exhibited statistically significant change, respectively, as detailed below.

### 2.1. Differentially Expressed miRNAs in Glucocorticoid Secreting Adrenal Adenoma Tissues

In adrenal tissue samples obtained from patients with active Cushing’s syndrome, five differentially expressed miRNAs were identified. MiR-375-3p, miR-566 and miR-210-3p were downregulated, while miR-95-3p and miR-506-3p were upregulated in cortisol-producing tissues compared to normal adrenals (Table 1). Investigating their potential function by pathway analysis of among others biotin metabolism, insulin signaling, adipocytokine pathway, Wnt and TGF-beta signaling were identified as regulated by these miRNAs (Table 2).

### 2.2. Expression Change of miRNAs in H295R and HeLa Cell Lines after Dexamethasone Treatment

Cell lines derived from different tissue origin cultured in steroid hormone-free complete media were used to investigate miRNA expressional change upon dexamethsone treatment. In HeLa cells, seven upregulated and one downregulated miRNA were identified compared to vehicle treatment (Table 1). Based on the bioinformatics analysis, these miRNAs regulate PI3K-Akt/mTOR, Wnt and TGF-beta signaling pathways (Table 2).

Dexamethasone treatment of H295R cells led to the upregulation of four and to the downregulation of two miRNAs compared to control cells (Table 1). The most significant pathways that are regulated in H295R cells by miRNAs upon steroid effect were focal adhesion, Wnt and ErbB signaling pathways (Table 2).

### 2.3. Common Pathways Regulated by Hypercortisolism through Different miRNAs in Different Cell Types

**Figure 1:**
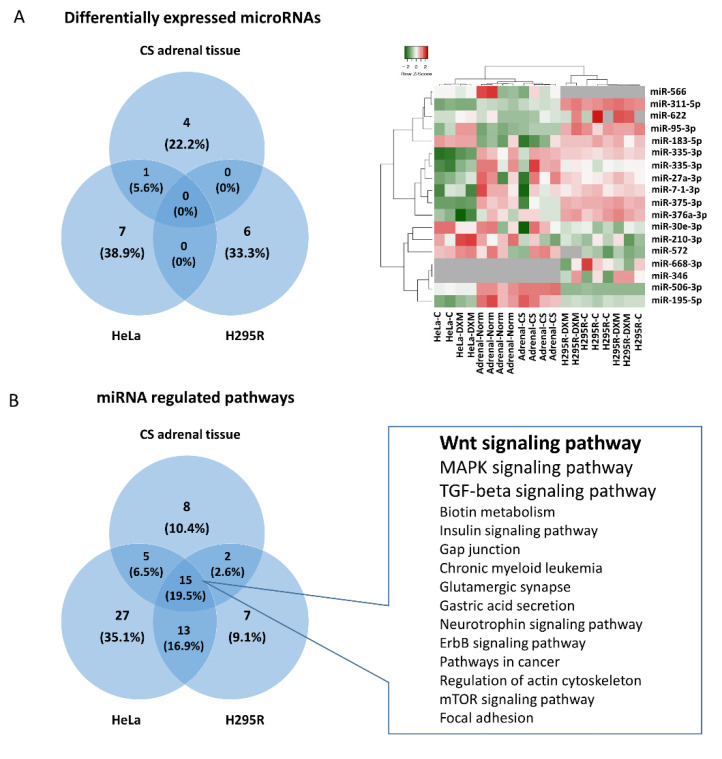
Differentially expressed miRNAs and the influenced signaling pathways upon hypercortisolism. Differential expression of only one common miRNA was detected among the experiments: miR-95-3p in cortisol producing adenomas and HeLa cells (**A**). Nonetheless, 19.5% of the significant signaling pathways regulated by different miRNAs identified in the separate experiments were common (**B**).

Altogether 18 miRNAs were detected to be differentially expressed upon increased cortisol effect in our three separate experiments (Figure 1A), interestingly, only one miRNA was commonly changed among the different groups: miR-95-3p was upregulated in both cortisol secreting adenomas and HeLa cells compared to their controls (Figure 1A). The differently expressed miRNAs regulated 30 signaling pathways in adrenal tissue, 60 enriched pathways in HeLa cells, and 37 significant pathways in H295R cells (*p* < 0.01) (Table 2). Despite the lack of common differentially expressed miRNAs among the cell types, a remarkable overlap was observed regarding the pathways influenced by these miRNAs. Fifteen pathways (19.5% of all) were commonly affected in all three experiments (Figure 1B). Functional bioinformatic analysis also revealed that of these 15 signaling pathways, Wnt-signaling seemed to be the most important followed by TGF-beta and MAPK signaling which could play a significant role in mediating hypercortisolism (Figure 1B).

### 2.4. Gene Expression Profiling of GC Producing and GC Target Tissue Types

Literature data mining and the re-analysis of the global transcriptome changes in GC producing and glucocorticoid target organs/cell types were performed. High-throughput transcriptome experiments of bone tissue originating from Cushing patients and primary bone, synovial fibroblast, subcutaneous and omental adipose tissue, skin and brain cells treated with dexamethasone (see in Section 4) were evaluated. Altogether, expressional data from 82 control and 57 GC affected samples were re-analyzed. The number of altered genes depending on tissue types varied between 1125 and 6809 (Table 3). While GC had low impact on global transcriptome changes on GC producing adrenal (4–8% of all genes profiled was changed), in GC target tissues, significant alteration was observed in roughly 10–20% of the expressed genes (Table 3). The number of down- and upregulated genes were approximately on the same scale, however in human primary osteoblast-like cells (HObs) from bone explant, mostly downregulated genes were observed (Table 3).

### 2.5. Investigation of Global Gene Expression Alteration upon Glucocorticoid Effect on Wnt Pathway Alteration in GC Target Tissues

Gene set enrichment analysis was performed of the differentially expressed genes upon GC treatment (Tables S1 and S2). Interestingly, among other influenced biological processes, Wnt signaling was significantly altered in all tissue types (Table 4, Tables S1 and S2).

By detailed analysis of the genes participating in Wnt signaling, generally, a high-ratio of members of the pathway showed significant gene expression alteration (see ratio in Table 4 and detailed results in Table S3).

Additionally, Wnt signaling remained significant when differentially expressed genes upon GC effect in at least six different studies were submitted to gene set enrichment analysis (Table S4).

### 2.6. Tissue-Specific Target Prediction of Wnt Pathway in GC-Producing Adrenal Adenoma

In cortisol-producing adenoma tissue, we could perform tissue specific target prediction. We selected the Wnt pathway members whose expression was negatively correlated with targeting miRNAs and also predicted by target prediction algorithms. Using this approach four genes were identified that were most probably regulated by GC mediated miRNAs (Table 5).

## 3. Discussion

The miRNA expression pattern is characteristic to each cell type; therefore, some miRNAs can be used as potential biomarkers for different conditions as well. GCs have diverse effects on different tissues involving various molecules including miRNAs. Our in vitro data showed that GC excess had significant effects on miRNA expression profiles on all tested cell types. In this context, commonly regulated miRNAs in HeLa and GC-producing/target tissues can be considered important mediators of GC effect, as HeLa cells represent a valid epithelioid-type target model and several GC-related processes occur in these cell types. Studying the biological role of significantly altered miRNAs upon GC treatment in three models, different miRNAs had a convergent effect on the same signaling pathways. Of these pathways, Wnt signaling was identified as the most relevant.

**Figure 2:**
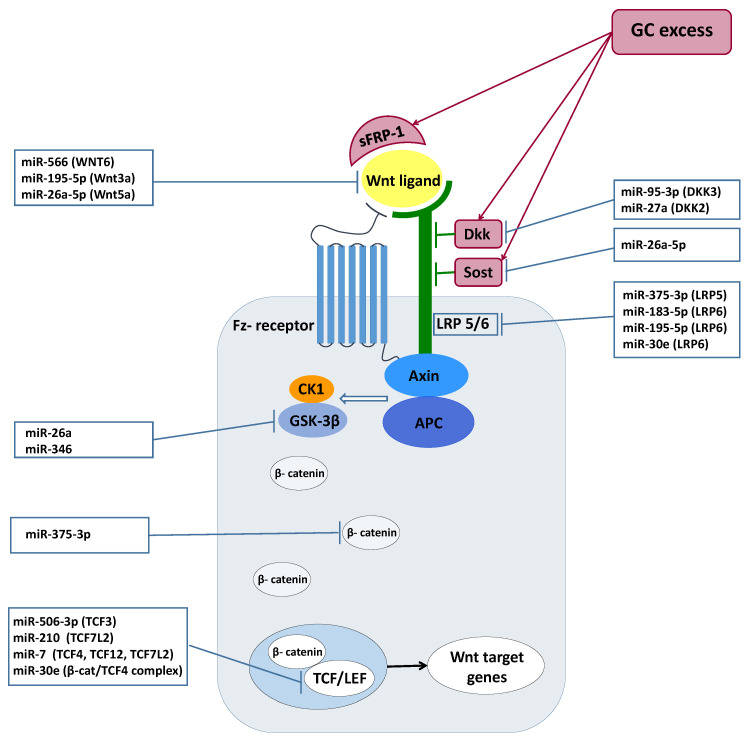
Wnt-pathway operation and the effect of the differentially expressed miRNAs [30,31,32,33,36,37,38,39,40,41,42,43,44,45,46,47,48,50,60].

The regulation of Wnt signaling through miRNAs has been studied and confirmed in several different tissues and cell types. Relying on literature data, we summarized the effect of the differentially expressed miRNAs identified in our experiments on the operation of the Wnt-signaling pathway. (Figure 2)

*GC effect on theGC-producing adrenal gland*. Interestingly, the tissue-specific target prediction indicated that even the cortisol-producing adrenal GCs have a feedback effect on miRNA expression that, as a consequence, influences Wnt signaling, among others. As expected, GC had a lower impact on the whole transcriptome in GC-producing cells and tissues compared to GC target tissues (4–8% of all genes changed vs. ~10–20%, respectively). It was described that miRNAs could play a role in regulating the operation of glucocorticoid receptors (GR) and consequently circadian rhythm [61]. The adrenal gland has its own peripheral circadian clock which is tightly linked to steroidogenesis by the steroidogenic acute regulatory protein [62]. These may give a potential explanation to the GC feedback effect in the adrenal gland.

Additionally, global transcriptome data indicated that in the adrenal gland the GCs regulated the Wnt pathway. Indeed, using both miRNA and gene expression data we performed tissue specific target prediction that indicated that in adrenal tissue miR-375 and miR-506 regulated *SFRP4*, *APC*, *NFAT5* and *CTBP2* expression among Wnt signaling members upon GC effect.

In the adrenal tissues obtained from hypercortisolic patients, the Wnt-signaling pathway was ranked backward compared to in vitro cell line experiments. Here, Wnt-signaling is preceded by *Biotin metabolism*, *Long-term depression*, *Insulin signaling pathway* and *Adipocytokine signaling pathway*. Not surprisingly, these pathways define some of the characteristic symptoms of Cushing’s disease including obesity, insulin resistance, depression, fatigue, irritability, insomnia, hair loss, dry skin [63,64]. This draws the attention to evaluate the results obtained on cell lines carefully as they miss the complex environment which is available in tissue experiments and in in vivo environments.

*GC effect on the GC-target cells/tissues*. In order to investigate the potential involvement of Wnt signaling in the GC effect on GC target tissues, we re-analyzed the data available in data repositories collected from bone tissue originating from Cushing patients and primary bone, synovial fibroblast, subcutaneous and omental adipose tissue, skin and brain cells upon GC effect. We found that the ratio of transcriptome regulated by GC depended on tissue type. Our results were in line with previous findings that in skin a relatively smaller gene set (6.3%) while in adipose tissue, brain and peripheral blood mononuclear cells a larger gene set (10–20%) were regulated by GC, [21,58,65,66]. Interestingly, in the liver GC induced vast transcriptional responses, with more than 30% of genes being regulated [59]. As we focused on Wnt signaling we investigated its involvement in whole transcriptome changes by three approaches (i) gene set enrichment analysis of global gene expression profile, (ii) gene set enrichment analysis of commonly changed gene profile at least in six different studies and (iii) analysis of individual Wnt pathways. In all approaches Wnt signaling was significantly altered upon GC effect and additionally we identified several miRNAs implicated in this pathway.

*miRNAs influencing Wnt signaling*. The identified GC-mediated miRNAs were demonstrated to influence Wnt signaling at different levels. Regarding the *Wnt ligands*, miR-195 and miR-26a-5p, suppressed the Wnt/β-catenin pathway among others through repressing *WNT3a, WNT5a*, and *WNT7a* in osteogenic differentiation of mesenchymal stem cells and periodontal ligament cells [38,39,41]. *WNT6* was also proved to be a target of miR-566 in human breast cancer [31]. *Wnt co-receptors* were also described as being controlled by GC-regulated miRNAs. LDL receptor-related proteins 5/6 (*LRP5* and 6) as co-receptors are indispensable for the activation of the canonical pathway. It was shown that miR-375-3p decreased the levels of *LRP5* and β-catenin by directly binding to their 3′UTR, therefore negatively regulating Wnt signaling [30]. Similarly, miR-183, by targeting *LRP6*, inhibited Wnt/β-catenin signaling pathway and consequently promoted adipogenesis [36]. Among *Wnt agonists*, *DKK3* was demonstrated to be regulated by miR-183 in prostate cancer [37]. miR-95-3p promoted cell proliferation, migration and invasion in PCa by targeting also *DKK3* and activating the Wnt/β-catenin pathway [32]. Additionally, *DKK2*, which is another direct inhibitor of Wnt binding to *LRP5/6*, was negatively regulated by miR-27a in in vitro miRNA overexpression and inhibition experiments [48]. *Wnt pathway mediators* are also regulated by GC-affected miRNAs. While the Wnt pathway effector *TCF7L2* was regulated by miR-7 leading to inhibition of Wnt/β-catenin signaling in neuronal cells [47], *TCF3*, another Wnt mediator, was repressed by miR-506-3p, resulting in neural stem cell proliferation and differentiation [33].

*The role of GC mediated miRNAs on biological processes*. Regarding the potential function of miRNAs altered upon GC effect, current literature data suggested that Wnt-signaling can be associated with cell proliferation and differentiation, tumorigenesis and bone homeostasis.

*Cell proliferation and differentiation.* MiR-506-3p upregulated in Cushing adenomas compared to normal tissues. In neural stem cells, the overexpression of this miRNA increased the cells’ differentiation and reduced their proliferation through influencing Wnt/β-catenin pathway by targeting *TCF3* [33]. In dermal papilla cells, miR-195-5p, by targeting LRP6 protein expression, influenced hair follicle inductivity [38]. The overexpression of miR-195-5p significantly inhibited osteogenic differentiation of periodontal ligament cells under mechanical loading together with regulating directly the WNT family member 3A (*WNT3A*) [39]. Upregulated miR-195-5p following GC treatment could suggest that this miRNA contributed to the bone damage and hair loss frequently observed in Cushing’s syndrome.

*Tumorigenesis.* In Cushing adenomas, as in a high glucocorticoid environment, we detected decreased miR-566 level. In breast cancer patients, the low expression of this miRNA predisposed to larger tumor size, advanced tumor grade and higher incidence of lymphatic metastasis [31]. MiR-566 represents an important link in this process by targeting *WNT6* and preventing malignant progression in breast cancer patients [31]. miR-95-3p, that was described upregulated in prostate carcinoma tissues, promoted cell proliferation, migration and invasion of cancerous cells through repressing *DKK3* prostate [32]. miR-183-5p expression was reported significantly upregulated in colorectal cancer tissues compared to normal tissues [67,68]. Its role in Wnt regulation was demonstrated by pathway analysis and functional miR-inhibition experiments that also led to the downregulation of the Wnt pathway downstream genes [67,68].

*Bone homeostasis.* Bones and bone-forming mechanisms are severely damaged by GC excess and a number of miRNAs have been identified that play important roles during these processes. Indeed, we presented the hypothesis that the expression profile of both human bone tissue biopsies from CS patients and patient-derived primary osteoblast cell lines treated by dexamethasone indicated that the Wnt pathway was significantly influenced by miRNAs. We also suggested that several GC-regulated miRNAs were demonstrated to be targeting Wnt signaling, including miR-375-3p, miR-210-3p and miR-26a-5p. MiR-375-3p was described to negatively modulate osteogenesis by inducing apoptosis in the mouse osteoblastic MC3T3-E1 cell line through targeting *LRP5* and β-catenin [30]. MiR-26a-5p also played a negative role by hindering the osteogenic differentiation of adipose-derived mesenchymal stem cells. This effect is accomplished directly, through the inhibition of *WNT5a* expression [41]. Overexpression of miR-210-3p promoted osteogenic differentiation and inhibited adipogenic differentiation in bone marrow-derived mesenchymal stem cells (BMSCs). In cells overexpressing miR-210 regulatory factors of the Wnt signaling pathway, such as *LRP5*, *GSK-3β*, β-catenin and *TCF4* were significantly increased [34]. The osteogenic effect of this miRNA was also demonstrated by downregulating the Wnt-pathway inhibitor sclerostin and by enhancing the migration capability of BMSCs [35]. In line with the in vitro results mentioned above, miR-210-3p was found to be downregulated in bone marrow samples of women suffering from postmenopausal osteoporosis [69]. In sphenoid bone samples of patients with active Cushing’s disease, the expression of miR-26a-5p was upregulated compared to patients with nonfunctioning pituitary adenoma [70].

Our results corroborate previous general knowledge about the function of miRNAs. Namely, that (i) miRNAs, similarly to mRNAs, have a tissue-specific expressional pattern [71], (ii) miRNAs exert a fine-tuning effect [72], and (iii) they work in network [73]. These three properties of miRNAs are also manifest in the mediation of the glucocorticoid effect. In different tissues different miRNA expressional patterns can be observed of the glucocorticoid effect, however, these differentially expressed miRNAs regulate similar biological processes in the different tissues, in particular, Wnt signaling.

## 4. Materials and Methods

### 4.1. Patients

Adrenal adenoma specimens from four patients with active Cushing’s syndrome were obtained during surgical interventions at Semmelweis University Department of Transplantation and Surgery. Normal adrenal samples from another four patients adjacent to hormonally inactive adrenal adenomas served as controls. The study was conducted in accordance with the Declaration of Helsinki and has been approved by the Scientific and Research Committee of the Medical Research Council of Hungary (ETT-TUKEB 4457/2012/EKU, approval date: 2 February 2012). All subjects gave written informed consent in accordance with the Declaration of Helsinki.

### 4.2. In Vitro Cell Culture Experiments

HeLa human epithelioid cervix cells were cultured in MEM (Gibco, 31095029, Thermofisher Scientific, Waltham, MA, USA) supplemented with 10% fetal bovine serum (Gibco 10270106, Thermofisher Scientific, Waltham, MA, USA), 1% sodium-pyruvate (Gibco, 11360070, Thermofisher Scientific, Waltham, MA, USA), and 1% antibiotic-antimycotic solution (Sigma-Aldrich, Merck, Kenilworth, NJ, USA). H295R cells were grown in Dulbecco’s Modified Eagle Medium Nutrient Mixture F-12 (Gibco: 10565018, Thermofisher Scientific, Waltham, MA, USA) supplemented with 2.5% Nuserum (Corning 355100, Corning, NY, USA), 1% Insulin-Transferrin-Selenium solution (41400-045 Gibco, Thermofisher Scientific, Waltham, MA, USA) and 1% antibiotic-antimycotic solution (Sigma-Aldrich, Merck, Kenilworth, NJ, USA). Cells were cultivated in a humidified incubator infused with 5% CO_2_ at 37 °C.

Before treatment, cells were grown in their complete media using hormone-free fetal bovine serum for 48 h. Hormone-free FBS were prepared by incubating and mixing 0.1 g dextran-coated active charcoal (C6241, Sigma-Aldrich, Merck, Kenilworth, NJ, USA) per 6 mL FBS for 24 h at 4 °C. After 24 h, mixtures were centrifuged 300× *g* for 10 min until charcoal settled and supernatant was filtered through 0.22 μm filter.

A measure of 100 nM dexamethasone (D4902, Sigma-Aldrich, Merck, Kenilworth, NJ, USA) or dimethyl sulfoxide (DMSO, 276855, Sigma-Aldrich, Merck, Kenilworth, NJ, USA) as vehicle treatment was applied on both cell lines for 24 h.

### 4.3. Gene Expression Profiling and Gene Expression Re-Analysis

Investigating transcriptome changes in GC-producing (adrenal tissue and cell line) and target organs (bone, synovial fibroblast, subcutaneous and omental adipose tissue, skin and brain) high-throughput mRNA profiling data (gene expression microarray, RNA sequencing and RT-PCR array [51,52,53,54,55,56,57,58,74]) were downloaded from NCBI Gene Expression Omnibus (Table 6). Altogether, data of 11 studies (82 control samples and 57 GC effected samples) were included. In order to reduce bias originating from usage of different platforms, different analysis type and cut-offs, we re-analyzed data using the same bioinformatical process, GEO2R algorithm with default settings and Benjamini−Hochberg (False discovery rate) adjustment [75].

### 4.4. RNA Extraction

Tumor tissue specimens were immediately frozen in liquid nitrogen after the surgery and stored at −80 °C until further use. From both tissues and cell cultures total RNA was extracted with miRNeasy Mini Kit (Qiagen Inc., Chatsworth, Los Angeles, CA, USA). RNA integrity and concentration were measured using Agilent Bioanalyzer 2100 System (AgilentTech Inc., Santa Clara, CA, USA).

### 4.5. miRNA Profiling in Adrenocortical Tissues Using TLDA Cards

All procedures were performed following the manufacturer’s instructions and as previously described [76]. Briefly, 900 ng total RNA per sample was reverse transcribed using Megaplex RT primer Pool A and B using TaqMan MicroRNA Reverse Transcription Kit (P/N: 4366596). MiRNA expression profiles were investigated using TaqMan Low Density Array (TLDA) Human Micro RNA Panel v.1 (Applied Biosystems, Foster City, CA, USA) on 7900HT Fast Real-Time PCR System (Applied Biosystems).

RT–qPCR data analysis was performed using RQ Manager 1.2 (Applied Biosystems). Real-Time StatMiner software (Integromics, Granada, Spain) were used to assess best endogenous control using Normfinder algorithm. (Normfinder calculates the overall stability for all candidate housekeeping genes tested on a sample set indifferent from its composition. It generates an overall stability rank from distinct intragroup and intergroup measures of variability). RNU48 for adrenal tissues and HeLa cells, the geometric mean of MammU6-RNU44-RNU6B for H295R were used as endogenous controls. Expression level was calculated by the ddCt method, and fold changes were obtained using the formula 2^−ddCt^.

### 4.6. Gene Set Enrichment and Pathway Analysis

Differentially expressed miRNAs were uploaded into the DIANA-mirPath v.2.0 tool. In silico target predictions were performed by microT-CDS algorithm and followed by enrichment analysis of multiple miRNA target genes comparing each set of miRNA targets to all known KEGG pathways [77].

Differentially expressed gene functions were analyzed using ToppGene Suite. Gene set enrichment analyses were performed for Gene Ontology categories (Biological Processes, Molecular Function and Cell Component) and KEGG Pathways gene sets. Results were considered to be significant with *p* < 0.05.

### 4.7. Statistical Analysis

Differences between Cushing vs. normal and dexamethasone treatment vs. DMSO treated cells were evaluated using unpaired T-test or Mann–Whitney U test depending on data distribution determined by Shapiro–Wilks normality test. Statistical analysis was performed using Integromics RealTime StatMiner and Statistica 13.4.0.14 (TIBCO Software Inc., Palo Alto, CA, USA).

Hierarchical cluster analysis was performed by UPGMA clustering method using Euclidean distance for similarity measuring. A value of *p* < 0.05 was considered to be significant.

## 5. Conclusions

Our results and literature data suggest that Wnt-signaling may be one of the most important pathways affected by hypercortisolism. It is, at least in part, regulated by miRNAs that mediate the glucocorticoid effect. Interestingly, in different cell types different miRNAs lead to the regulation of very similar pathways, including Wnt-signaling. In the in vitro cell culture experiments and in GC-secreting adrenal tissues, the Wnt signaling was among the primarily affected pathways upon hypercortisolic effect. The results and transcriptome data obtained from GC target tissues suggest that the alteration of Wnt signaling (together with other signaling pathways) may be responsible for the leading symptoms observed in CS.

## Figures and Tables

**Table 1:** Differentially expressed miRNAs in GC-secreting adrenal adenoma, in HeLa and H295R cells after glucocorticoid treatment and their connection with the elements of Wnt-signaling pathway (*—sclerostin domain-containing 1, **—based on pathway analysis Wnt-signaling is between the top targeted pathways). Log2FC indicates the direction and the extent of expressional change.

miRNA Name	log2FC	*p*-Value	Connection with Wnt-Signaling	
**Cortisol Producing Adenoma vs. Normal Adrenal Tissue**			**Targeted Gene(s)**	**References**
hsa-miR-375-3p	−3.18283	0.002756	LRP5 and β-catenin	[30]
hsa-miR-566	−2.0934	0.012993	WNT6	[31]
hsa-miR-95-3p	0.435871	0.014638	DKK3	[32]
hsa-miR-506-3p	1.96571	0.021431	TCF3	[33]
hsa-miR-210-3p	−1.18389	0.038611	WNT7b, FZD5, Sclerostin	[34,35]
Dexamethasone Treated HeLa Cells vs. Control				
hsa-miR-183-5p	0.203726	0.037677	LRP6, Dkk-3	[36,37]
hsa-miR-195-5p	1.355332	0.043846	LRP6, WNT3A	[38,39]
hsa-miR-26a-5p	1.321971	0.020827	SOSTDC1 *, Wnt5a, GSK3β	[40,41,42]
hsa-miR-30e-3p	−0.86986	0.02123	LRP6	[43]
hsa-miR-335-3p	1.586908	0.045771	RUNX2	[44]
hsa-miR-572	1.58437	0.042381	pathway analysis **	[45]
hsa-miR-622	3.287344	0.03071	pathway analysis **	[46]
hsa-miR-95-3p	2.307738	0.040524	DKK3	[32]
Dexamethasone Treated H295R Cells vs. Control				
miR-331-5p	2.14926	0.018581	no literature data available	
miR-7-1-3p	−0.25662	0.004913	TCF4, TCF12, TCF7L2	[47]
miR-27a-3p	0.457272	0.010971	DKK2	[48]
miR-346	0.921683	0.017707	GSK3β	[49]
miR-376a-3p	0.581655	0.034872	pathway analysis **	[50]
miR-668-3p	−0.56929	0.041363	no literature data available	

**Table 2:** Pathways regulated by differentially expressed miRNAs among different experiments. Common pathways are indicated by bolt letters.

GC-Secreting Adrenal Adenoma		Hela Cells		H295R Cells	
**KEGG Pathway**	***p*-Value**	**KEGG Pathway**	***p*-Value**	**KEGG Pathway**	***p*-Value**
Biotin metabolism	5.90E-06	PI3K-Akt signaling pathway	1.87E-26	Focal adhesion	5.71E-09
Axon guidance	5.90E-06	Prostate cancer	5.59E-20	**Wnt signaling pathway**	4.44E-08
Long-term depression	3.95E-04	**Wnt signaling pathway**	8.18E-14	Neurotrophin signaling pathway	4.44E-08
Insulin signaling pathway	3.23E-03	mTOR signaling pathway	5.65E-13	ErbB signaling pathway	1.08E-07
Gap junction	7.57E-03	Insulin signaling pathway	1.74E-12	Gap junction	1.08E-07
Chronic myeloid leukemia	8.75E-02	Focal adhesion	2.14E-11	PI3K-Akt signaling pathway	1.08E-07
Glutamatergic synapse	1.84E-01	Ubiquitin mediated proteolysis	8.16E-11	GnRH signaling pathway	1.85E-06
Adipocytokine signaling pathway	1.84E-01	**TGF-beta signaling pathway**	1.03E-10	Circadian rhythm	2.06E-05
**Wnt signaling pathway**	2.65E-01	Pathways in cancer	5.91E-10	**MAPK signaling pathway**	2.06E-05
Gastric acids secretion	2.65E-01	Melanoma	1.32E-09	Ubiquitin mediated proteolysis	3.30E-05
Salmonella infection	2.91E-01	p53 signaling pathway	1.69E-09	Glioma	5.05E-05
Neurotrophin signaling pathway	3.95E-01	Regulation of actin cytoskeleton	1.02E-08	Long-term potentiation	1.06E-04
**MAPK signaling pathway**	5.45E-01	Glioma	2.31E-08	Pathways in cancer	2.11E-04
Retrograde endocannabinoid signaling	6.21E-01	Long-term potentiation	2.58E-08	Insulin signaling pathway	2.58E-04
ErbB signaling pathway	6.21E-01	Long-term depression	2.76E-08	mTOR signaling pathway	2.08E-03
**TGF-beta signaling pathway**	6.77E-01	Gap junction	4.83E-08	Axon guidance	2.45E-03
Pathways in cancer	7.03E-01	Endometrial cancer	4.83E-08	Dopaminergic synapse	4.01E-03
Acute myeloid leukemia	9.43E-01	Dopaminergic synapse	5.80E-07	Small cell lung cancer	5.79E-03
HTLV-I infection	9.76E-01	Fatty acid biosynthesis	5.87E-07	Lysine degradation	6.25E-03
GnRH signaling pathway	1.34E+00	Neurotrophin signaling pathway	6.26E-07	Protein processing in endoplasmic reticulum	9.83E-03
Shigellosis	1.48E+00	**MAPK signaling pathway**	8.51E-07	Prostate cancer	2.00E-02
Regulation of actin cytoskeleton	1.86E+00	Chronic myeloid leukemia	4.10E-06	**TGF-beta signaling pathway**	2.13E-02
Pancreatic cancer	2.43E+00	HIF-1 signaling pathway	6.81E-06	Amphetamine addiction	6.20E-02
Maturity onset diabetes of the young	3.57E+00	ErbB signaling pathway	7.23E-06	Hedgehog signaling pathway	9.27E-02
Cholinergic synapse	4.00E+00	Small cell lung cancer	1.07E-05	Chronic myeloid leukemia	1.13E-01
mTOR signaling pathway	4.04E+00	Non-small cell lung cancer	4.37E-05	RNA transport	1.54E-01
Focal adhesion	4.79E+00	Acute myeloid leukemia	5.51E-05	Melanogenesis	2.37E-01
Pancreatic secretion	4.79E+00	Prion diseases	4.79E-04	Regulation of actin cytoskeleton	4.01E-01
Non-small cell lung cancer	5.54E+00	mRNA surveillance pathway	6.30E-04	Renal cell carcinoma	6.54E-01
Salivary secretion	7.96E+00	Transcriptional misregulation in cancer	1.48E-03	Glutamatergic synapse	6.54E-01
		Gastric acid secretion	3.44E-03	p53 signaling pathway	9.56E-01
		Calcium signaling pathway	6.18E-03	Gastric acid secretion	1.39E+00
		Aldosterone-regulated sodium reabsorption	8.44E-03	Biotin metabolism	1.63E+00
		Hedgehog signaling pathway	1.28E-02	Transcriptional misregulation in cancer	2.47E+00
		T cell receptor signaling pathway	1.83E-02	Bacterial invasion of epithelial cells	4.44E+00
		RNA transport	2.76E-02	Fc gamma R-mediated phagocytosis	7.41E+00
		Glutamatergic synapse	3.14E-02	Fc epsilon RI signaling pathway	7.97E+00
		Renal cell carcinoma	4.60E-02		
		Oocyte meiosis	4.81E-02		
		Cell cycle	4.89E-02		
		Biotin metabolism	6.21E-02		
		Colorectal cancer	1.11E-01		
		Basal cell carcinoma	2.68E-01		
		Inositol phosphate metabolism	2.68E-01		
		VEGF signaling pathway	2.68E-01		
		B cell receptor signaling pathway	2.68E-01		
		Pancreatic cancer	2.68E-01		
		Cholinergic synapse	3.50E-01		
		Melanogenesis	3.72E-01		
		Arrhythmogenic right ventricular cardiomyopathy (ARVC)	1.19E+00		
		Hypertrophic cardiomyopathy (HCM)	1.21E+00		
		Progesterone-mediated oocyte maturation	1.21E+00		
		Apoptosis	1.52E+00		
		Tight junction	1.52E+00		
		Thyroid cancer	1.52E+00		
		Viral carcinogenesis	2.40E+00		
		Phosphatidylinositol signaling system	2.41E+00		
		Bladder cancer	2.65E+00		
		Cocaine addiction	3.14E+00		
		Fanconi anemia pathway	3.92E+00		

**Table 3:** Transcriptome changes of GC producing and GC target tissue types.

Gene Expression Study	Time-Dex Treatment	Dose-Dex Treatment	All Genes Measured	# of Not Regulated Genes	# of DEGs (*p* < 0.05)	Ratio of Regulated Genes	# of Upregulated Genes	# of Downregulated Genes
Adrenal adenoma vs. normal cortex [51]	na	na	41,078	37,522	3556	0.08	1631	1925
Human ACC cancer cell line (H295R) control vs. dex [52]	6 h	100 nM	29,153	27,886	1267	0.04	585	682
Human bone tissue biopsies from CS patients, before vs. 3 months after surgery [53]	na	before and mean 3 months after surgery	54,675	53,417	1258	0.02	608	650
Human primary osteoblast cell control vs. dex [54]	24 h	100 nM	22,177	16,834	5343	0.24	2683	2660
Human primary osteoblast-like cells (HObs) from bone explant control vs. dex [55]	24 h	100 nM	54,675	47,866	6809	0.12	199	6610
Human primary synovial fibroblasts control or dex * [56]	24 h	100 nM	81 *	74 *	7 *	0.08 *	6 *	1 *
human primary abdominal subcutaneous adipose cells control vs. dex [57]	7 days	1000 nM	19,741	15,465	4276	0.21	2130	2146
Human primary abdominal omental adipose cells control vs. dex [57]	7 days	1000 nM	19,741	15,954	3787	0.19	2091	1696
Human primary epidermal keratinocytes control vs. dex [58]	24 h	100 nM	12,625	11,500	1125	0.08	656	469
Piglet hippocampus tissue control vs. im. dex treated [59]	3 h	60 µg/kg	16,764	14,361	2403	0.14	1156	1247
Piglet hypothalamus tissue control vs. im. dex treated [59]	3 h	60 µg/kg	16,764	14,103	2661	0.15	1311	1350

DEGs: differentially expressed genes; dex: dexamethasone; ACC: adrenocortical cancer; CS: Cushing’s syndrome; na: not applicable; *: due to the smaller scale custom platform used in this experiment, data should be considered accordingly. #: The number of DEGs.

**Table 4:** Wnt signaling is significantly altered upon GC effect in all experiments.

GC Target Tissue Type’s Gene Expression	# of DEGs (*p* < 0.05)	Ratio of WNT Pathway Members *	# of Affected BP	# of Affected BP Related to WNT Signaling **
Human bone tissue biopsies from CS patients, before vs. 3 months after surgery [53]	1423	14/151	312	4
Human primary osteoblast cell control vs. dex [54]	5343	52/151	1573	11
Human primary osteoblast-like cells (HObs) from bone explant control vs. dex [55]	7279	53/151	1164	6
Human primary synovial fibroblasts control or dex [56]	10	8/151	880	41
Human primary abdominal subcutaneous adipose cells control vs. dex [57]	4276	37/151	2644	16
Human primary abdominal omental adipose cells control vs. dex [57]	3787	32/151	2422	6
Human primary epidermal keratinocytes control vs. dex [58]	1130	13/151	1753	7
Piglet hippocampus tissue control vs. im. dex treated [59]	2403	34/151	1913	10
Piglet hypothalamus tissue control vs. im. dex treated [59]	2661	33/151	1850	11

*: ratio of WNT pathway members: differentially expressed gene in Wnt signaling/all members (151 genes) of Wnt pathway according to KEGG gene set (hsa04310); **: detailed BP categories are listed in Table S2; #: The number of DEGs; BP: biological process (gene ontology category); DEGs: differentially expressed genes, dex: dexamethasone; CS: Cushing’s syndrome.

**Table 5:** Tissue-specific target prediction of miRNAs differentially expressed in human cortisol-producing adrenal adenoma tissues.

Targeting miRNA	Gene Symbol	Gene Title	logFC	*p*-Value	GSE14922 Probe ID
miR-375	SFRP4	Secreted frizzled related protein 4	1.3979	0.0077694	A_23_P215328
miR-506	APC	APC, WNT signaling pathway regulator	−2.1794	0.0278861	A_23_P70213
miR-506	NFAT5	Nuclear factor of activated T-cells 5	−1.0592	0.0343637	A_23_P359647
miR-375	CTBP2	C-terminal binding protein 2	0.9465	0.037437	A_23_P63897

**Table 6:** Gene expression profiling of glucocorticoid (GC) producing and target tissue types. (CS: Cushing syndrome, cortisol-producing adrenal adenoma; dex: dexamethasone; #: the munber of Controls).

Sample Type	Experiment	# of Control	# of Dex Treatment/GC Affected	DataSet [Refs]	Platform
Human tissue	bone tissue biopsies from CS patients, before vs. 3 months after surgery	9	9	GSE30159 [53]	Affymetrix Human Genome U133 Plus 2.0 Array
Human tissue	adrenal cortex, normal tissue vs. GC secreting adenoma	4	4	GSE14922 [51]	Agilent-014850 Whole Human Genome Microarray 4 × 44 K G4112F
In vitro human cell line	adrenocortical cancer (H295R) cell line, control vs. dex treatment	3	3	GSE64826 [52]	Affymetrix Human Gene 1.0 ST Array
In vitro human primary cells	osteoblast cell, control vs. dex	6	3	GSE21727 [54]	Illumina humanRef-8 v2.0 expression bead chip
In vitro human primary cells	osteoblast-like cells (HObs) from bone explant, control vs. dex treatment	6	3	GSE10311 [55]	Affymetrix Human Genome U133 Plus 2.0 Array
In vitro human primary cells	synovial fibroblasts, control or dex treatment	6	3	GSE37520 [56]	Applied Biosystems/University of Birmingham Human RT-PCR array (custom made)
In vitro human primary cells	abdominal subcutaneous adipose cells, control vs. dex treatment	3	3	GSE88966 [57]	Affymetrix Human Gene 1.0 ST Array
In vitro human primary cells	abdominal omental adipose cells, control vs. dex treatment	3	3	GSE88966 [57]	Affymetrix Human Gene 1.0 ST Array
In vitro human primary cells	epidermal keratinocytes, control vs. dex treatment	2	2	GSE26487 [58]	Affymetrix Human Genome U95 Version 2 Array
German Landrace piglet tissue	7-week old purebred piglet hippocampus tissue, control vs. im. dex treatment	20	12	Murani et al., 2021 [74]	TruSeqStranded mRNA sample preparation kit; HiSeq 2500 instrument
German Landrace piglet tissue	7-week old purebred piglet hypothalamus tissue, control vs. im. dex treatment	20	12	Murani et al., 2021 [74]	TruSeqStranded mRNA sample preparation kit; HiSeq 2500 instrument

## Data Availability

All data are presented in the manuscript.

## Supplementary Materials

The following are available online at https://www.mdpi.com/article/10.3390/ijms222111778/s1.
